# Ship–Bridge Collision Real-Time Alarming Method Based on Cointegration Theory

**DOI:** 10.3390/s25051488

**Published:** 2025-02-28

**Authors:** Wanwen Zhong, Deling Liu, Chunhui Xie, Kuijun Zhang, Wenkai Zhan, Maosen Cao, Yufeng Zhang

**Affiliations:** 1 College of Mechanics and Engineering Science, Hohai University, Nanjing 211100, China; zhongwanwen@hhu.edu.cn (W.Z.); chunhuixie@hhu.edu.cn (C.X.); cmszhy@hhu.edu.cn (M.C.); 2 Quality and Safety Center for Water Resources Engineering of Shandong Province, Jinan 250013, China; liudeling_73@163.com (D.L.); zhangkj@shandong.cn (K.Z.); 15854189919@163.com (W.Z.); 3 State Key Laboratory of Safety, Durability and Healthy Operation of Long Span Bridges, Jiangsu Transportation Institute Group Co., Ltd., Nanjing 210017, China; 4 Jiangsu Transportation Institute Group Co., Ltd., 2200 Chengxin Avenue, Jiangning District, Nanjing 210017, China

**Keywords:** ship–bridge collision, alarming technology, cointegration, Kalman filter, damage index

## Abstract

Ship–bridge collisions in inland waterways pose a serious threat to bridge infrastructure, often resulting in structural damage and jeopardizing safety. Despite the widespread deployment of collision warning systems, these systems fail to function effectively due to factors such as weather conditions, equipment malfunctions, and human error. Current alarming technologies, such as wavelet-based methods, are limited by poor real-time performance, high sensitivity to noise, and low localization accuracy, which hinder their practical application. This paper proposes an innovative Kalman filter–cointegration alarming (KFCA) technology, combining cointegration theory with Kalman filtering to achieve precise and real-time collision detection. Through numerical simulation, KFCA is validated, with the results summarized as follows: (i) KFCA effectively recognizes ship–bridge collisions under an SNR of 60, 70, and 80 dB; and (ii) it accurately identifies impact locations on the bridge based on sensor arrangement indices. Compared to existing methods, KFCA offers significant advantages in real-time response, noise resistance, and localization accuracy. This technology provides an efficient solution for bridge management departments, enabling the timely and accurate detection of ship–bridge collisions, thereby enhancing bridge safety and reducing secondary disasters.

## 1. Introduction

With the rapid development of shipping and bridge construction, the number of ship–bridge collision accidents is also increasing. Such accidents not only cause significant economic losses [1,2,3] but also pose threats to safety. For example, in 2024, the Lixinsha Bridge in Guangzhou was struck by a ship, resulting in the collapse of the bridge deck. Due to the lack of immediate alarm, five vehicles fell directly off the bridge within 7 min of the accident, causing five deaths. In the same year, a severe ship–bridge collision occurred in Baltimore, USA, demolishing a 1.6-mile-long steel bridge and causing six deaths.

To effectively mitigate the disaster risks associated with ship–bridge collisions, the concept of an early warning mechanism has been proposed. The core of this mechanism lies in the ability to issue early warning signals to relevant personnel through real-time monitoring and data analysis before a collision occurs between a ship and a bridge, enabling timely adjustment of the ship’s course to prevent accidents. Currently, the main early warning technologies include ship–bridge collision early warning systems based on Automatic Identification Systems (AISs) [4,5], machine learning [6,7,8,9], laser measurement technology [10], and radar technology [11]. The AIS, as a crucial database, provides comprehensive ship information but is limited by latency issues. Existing machine learning-based ship–bridge collision early warning systems for bridges typically require the integration of multi-sensor data to improve the accuracy of warnings. However, AIS and machine learning technologies face challenges, including the complexity of early warning system setup and the lack of real-time efficiency. Both laser and radar offer good recognition capabilities but are individually prone to weather conditions and electromagnetic interference. Moreover, expensive and complex technologies are challenging to deploy comprehensively on smaller ships. In summary, existing ship–bridge collision early warning technologies lack sufficient real-time performance and environmental interference resistance, making it difficult to provide ships with enough time to adjust their positions, thus resulting in suboptimal effectiveness in practice.

Early warning aims to prevent accidents and occurs before a ship–bridge collision. In contrast, an alarm focuses on the early identification of a collision event to mitigate secondary disasters, occurring after a ship–bridge collision. Recent research on real-time alarming technologies for ship–bridge collisions is relatively limited. Duan et al. [12] proposed a real-time ship–bridge collision alarming technology based on raw data collected by sensors, employing data change rates and Euclidean distance. Zhang et al. [13] identified three critical features of bridge horizontal end displacement—the horizontal angle amplitude at the span end, the range between the maximum and minimum values of the first mode displacement, and the amplitude of the power spectral density—which were used as safety indicators. They provided corresponding safety thresholds for these features to identify the timing of ship–bridge collisions. Zheng et al. [14] introduced a ship–bridge collision monitoring system based on flexible quantum tunneling composites with buffering capabilities, which can detect the occurrence time and duration of ship–bridge collisions by studying the materials’ collision sensing and localization abilities. Zhang et al. [15] introduced a technology for detecting bridge anomalies based on Long Short-Term Memory (LSTM) networks, using a small set of historical normal data to train the network, and then detecting ship–bridge collisions by evaluating the prediction errors from the trained network.

These existing methods have unique advantages in data-driven approaches, smart material applications, and deep learning. However, there is still room for improvement in (1) noise resistance, (2) applicability to bridges of different scales, (3) dependence on historical data, and (4) the real-time performance of alarming. Hence, it is essential to develop a real-time ship–bridge collision alarming technology with strong noise and environmental interference resistance and high real-time performance to reduce the severity of accidents. This paper introduces the cointegration concept from econometrics [16] and integrates it with the Kalman filter to develop a ship–bridge collision alarming technology that is designed to alert to the timing and location.

Unlike other structural health monitoring methods [17,18,19], the traditional principle of using cointegration for damage detection is to generate stationary residuals from nonstationary variables [20,21,22]. Damage can be detected by observing abnormal residual behavior [23,24], with the aim of reducing the interference of environmental factors. The Kalman filter, as a recursive algorithm, is well suited to online data processing and is widely used in real-time target tracking [25] and state estimation. The Kalman filter–cointegration [26] method applied in this paper integrates the strong resistance of cointegration methods to environmental factors with the real-time performance of the Kalman filter. On this basis, assuming that sensors are functioning properly [27], data are collected for computation, and safety thresholds are set to achieve the real-time identification of ship–bridge collisions. Furthermore, this paper proposes a new damage index based on cointegration theory to determine the location of ship impacts on bridge piers. In light of the above, KFCA is proposed, which combines the timing of ship–bridge collisions with impact locations.

The KFCA proposed in this paper addresses several issues with existing ship–bridge collision alarming technologies. (1) Poor noise resistance: the cointegration theory in KFCA effectively reduces interference from environmental noise. (2) Limited applicability: KFCA does not require complex monitoring equipment and can analyze data collected by sensors, making it suitable for bridges of most scales. (3) Dependence on historical data: traditional machine learning-based alarming technologies require large amounts of historical data samples, whereas KFCA introduces an adaptive decay factor, increasing the weight of new data and reducing dependence on historical data. (4) Insufficient real-time performance: KFCA processes data using a sliding window approach, and the introduction of the adaptive decay factor enhances its sensitivity to newly introduced data. These improvements enhance real-time performance.

The structure of this paper is as follows: Section 2 introduces the fundamental concepts of cointegration and Kalman filtering. Section 3 describes the ship–bridge collision real-time alarming technology based on cointegration and Kalman filtering, along with the damage index used to identify the impact location. Finally, it summarizes the workflow. Section 4 presents the details of the numerical simulation for ship–bridge collisions. Section 5 uses the numerical simulation results to apply KFCA to ship–bridge collision alarming, taking noise factors into account. The effectiveness of the alarming technology is validated by comparing it with the conventional wavelet transform technology. The proposed damage index also successfully identifies the location of the ship impact, and its effectiveness is validated under various conditions. Finally, Section 6 concludes the paper with a summary.

## 2. Fundamental Concepts

### 2.1. Cointegration Theory

The stationarity of a time series is determined by its statistical properties, such as mean and variance. If a time series has statistical properties that do not change over time, it is said to be stationary; if the opposite is true, it is said to be nonstationary. In reality, most time series are nonstationary, but they can be converted into stationary series through differencing operations [28]. If a nonstationary time series $y_{t}$ becomes stationary after differencing the series d times, it is called an integrated series of order d, denoted $y_{t}$ ~ I(d). The Augmented Dickey–Fuller (ADF) test [29], widely used in economics, is employed to determine the order of integration of time series.

Time series analysis extends beyond studying individual series to include the examination of relationships among multiple time series. In this regard, the cointegration theory and method proposed by Engle and Granger [10] are used to describe the long-term equilibrium relationships among multiple nonstationary time series. Although some sequences of variables are nonstationary, specific linear combinations of these variables may be stationary. This process of linear combination is known as cointegration. Suppose there are n nonstationary time series ${\left[y_{1t},y_{2t},y_{3t},\ldots ,y_{nt}\right]}^{T}$; the linear combination of these series is written as follows:

(1) $$\beta^{T}\gamma_{t}=\beta_{1}y_{1t}+\beta_{2}y_{2t}+\cdot \cdot \cdot +\beta_{n}y_{nt}$$

where $\beta_{1},\beta_{2},...\;\beta_{n}$ are the coefficients of the cointegration equation, and $\beta^{T}$ is the cointegrating vector. Equation (1) contains n−1 linearly independent vectors. This linear combination $\beta^{T}\gamma_{t}$ is referred to as the cointegration residual.

In reality, the purpose of cointegration is to find the long-term equilibrium among nonstationary time series. Before equilibrium can be achieved, two key prerequisites need to be satisfied [30]. First, the nonstationary time series in cointegration analysis must have at least one common trend, and second, they must have the same order of integration. Once these conditions are met, the Johansen cointegration test [31] can be employed to build a Vector Error Correction Model, estimate [32] the cointegration vectors, and determine the long-term equilibrium relationships:

(2) $$\Delta y_{t}=\rho y_{t-1}+\sum_{i=1}^{m-1}\gamma_{i}\Delta y_{t-i}+\varepsilon_{t}$$

where $y_{t}$ a first-order integrated series I(1), and $\rho \in {\mathbb{R}}^{N\times N}$ is a rank-loss matrix of rank r<N.

If a cointegration relationship exists between the two monitored variables, the cointegration equation can be expressed as

(3) $$y_{i}(t)=a_{1}y_{j}(t)+a_{2}+\varepsilon (t)$$

where $y_{i}(t)$ and $y_{j}(t)$ are observed variables, $a_{1}$ and $a_{2}$ are the coefficients for the respective linear combination, which are also referred to as cointegration coefficients, and ε(t) is the stochastic disturbance term.

### 2.2. Kalman Filter

The Kalman filter algorithm [33] is a time-domain discrete autoregressive optimization algorithm widely used for predicting the position, velocity, and other state information of dynamic targets. The algorithm is based on the state space model of linear dynamic systems, which assumes that the system state evolves over time and is updated by noisy observations. The model defines the state vector x and the observation equation H. The Kalman filter essentially consists of two steps: prediction and measurement update. During the prediction update step, the algorithm uses the state vector from the previous time to predict the state at the next time step. During the measurement update step, the Kalman gain matrix is initially computed, and the state estimate and error covariance matrix at time t are updated using the observation variable $f_{j}(t)\;$:

Prediction update:

(4) $${\tilde{x}}_{k+1}={\hat{x}}_{k}$$

(5) $${\tilde{P}}_{k+1}={\hat{P}}_{k}+Q_{k}$$

Measurement update:

(6) $$K_{k+1}={\tilde{P}}_{k+1}H_{k+1}^{T}{\left(H_{k+1}{\tilde{P}}_{k+1}H_{k+1}^{T}+R_{k+1}\right)}^{-1}$$

(7) $${\hat{P}}_{k+1}=(I-K_{k+1}H_{k+1}){\tilde{P}}_{k+1}$$

(8) $${\hat{x}}_{k+1}={\tilde{x}}_{k+1}+K_{k+1}[y_{i(k+1)}-H_{k+1}{\tilde{x}}_{k+1}]$$

where ${\hat{x}}_{k}$ is the estimated value of $x_{k}$ at time kΔt, ${\tilde{x}}_{k+1}$ is the predicted value of $x_{k+1}$ at time (k+1)Δt, ${\hat{P}}_{k}$ and ${\tilde{P}}_{k+1}$ represent the error covariance matrices associated with ${\hat{x}}_{k}$ and ${\tilde{x}}_{k+1}$, I is an unit matrix, $y_{i(k+1)}$ is the observed variable, $tr\left[\;\right]$ represents the trace of the matrix, and $K_{k+1}$ is the Kalman gain matrix.

## 3. Kalman Filter–Cointegration Alarming (KFCA) Technology

### 3.1. Formulation of KFCA

A bridge operates in a nonstationary environment under normal conditions, and the cointegration theory can be applied to describe the long-term dynamic equilibrium relationship of the bridge system under nonstationary random excitations. Based on this, the Kalman filter algorithm is introduced, allowing the instant of a ship–bridge collision to be rapidly detected by the real-time tracking of changes in the cointegration coefficients. In this technology, the lateral displacements $y_{i}$ and $y_{j}$ collected from two sensors at level height i=8 are used for Johansen cointegration, and the resulting cointegration equation serves as the observation equation of the Kalman filter. The computed cointegration coefficients form the state vector, thereby establishing the corresponding state space model:

(9) $$\dot{x}(t)=\left\{\begin{array}{l} {\dot{a}}_{1}\\ {\dot{a}}_{2} \end{array}\right\}+\omega =0+\omega$$

(10) $$y_{i}=Hx+\varepsilon (t)$$

where $x={\left[a_{1}\;a_{2}\right]}^{T}$ is represented as the state vector composed of the cointegration coefficients, ω is the zero-mean stationary process noise with covariance Q, $y_{i}$ and $y_{j}$ are the lateral displacement data across the bridge, and ε is the observation noise with covariance matrix R. The observation matrix H can be represented as

(11) $$H=\left[y_{j}(t)\;1\right]$$

Discretizing Equations (9) and (10) over the time interval kΔt (with Δt as the sampling time step) yields the following discrete-time state model:

(12) $$x_{k+1}=x_{k}+\omega_{k}$$

(13) $$y_{i(k)}=H_{k}x_{k}+\varepsilon_{k}$$

For the discrete bridge response system described in Equations (12) and (13), the KFCA applied in this paper can be used to perform the real-time estimation of the cointegration relationship. An adaptive decay factor $\lambda_{k}$ is introduced to avoid over-reliance on historical bridge displacement data and to enhance the weight of recent observations in the system.

(14) $$Z_{k}=y_{i(k+1)}-H_{k}{\tilde{x}}_{k+1}$$

(15) $$\lambda_{k}=\max\left\{\;1\;,\frac{{Z_{k}}^{T}Z_{k}-tr\left[H_{k}Q_{k-1}H_{k}^{T}+R_{k}\right]}{tr\left[H_{k}{\hat{P}}_{k-1}H_{k}^{T}\right]}\right\}$$

Prediction update:

(16) $${\tilde{x}}_{k+1}={\hat{x}}_{k}$$

(17) $${\tilde{P}}_{k+1}=\lambda_{k}{\hat{P}}_{k}+Q_{k}$$

Measurement update:

(18) $$K_{k+1}={\tilde{P}}_{k+1}H_{k+1}^{T}{\left(H_{k+1}{\tilde{P}}_{k+1}H_{k+1}^{T}+R_{k+1}\right)}^{-1}$$

(19) $${\hat{P}}_{k+1}=(I-K_{k+1}H_{k+1}){\tilde{P}}_{k+1}$$

(20) $${\hat{x}}_{k+1}={\tilde{x}}_{k+1}+K_{k+1}\left[y_{i(k+1)}-H_{k+1}{\tilde{x}}_{k+1}\right]$$

Based on cointegration theory, the long-term stationary relationship between the data collected by the two sensors will be disturbed by the external factor of a ship collision, causing changes in the corresponding cointegration coefficients. The Kalman filter algorithm enables the real-time estimation of these cointegration coefficients, facilitating high real-time alarming performance. Additionally, this paper employs traditional Statistical Process Control [34] methods to establish the upper control limit (UCL) and lower control limit (LCL) for the alarming safety threshold. If the estimated cointegration coefficients surpass these thresholds, it is determined that a ship–bridge collision has occurred.

(21) $$UCL,LCL=CL\pm G\cdot \frac{\sigma }{\sqrt{j}}$$

where CL is the mean value of the cointegration coefficients estimated by the alarming technology, G is the standard normal distribution value corresponding to the confidence level (in this paper, the confidence level is 95%, so G = 1.96), and σ is the standard deviation of the estimated cointegration coefficients, while j is the sample size.

### 3.2. Impact Location Identification Index

**Figure 1:**
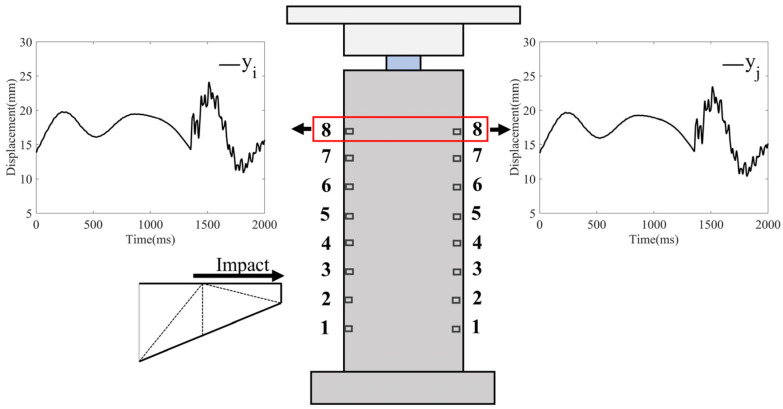
Ship–bridge collision and data collection.

The KFCA proposed in this paper can accurately identify the time of impact when a ship–bridge collision occurs and provides timely alarming information. On this basis, if the locations of ship–bridge collisions can be identified, it will further help bridge management departments to take emergency measures quickly, ensuring the safety of both the bridge and the ship. Therefore, based on the cointegration relationship between data collected from multiple sensors, this section proposes a new location identification index—the cointegration coefficient energy difference—to identify the location of ship–bridge collisions. As shown in Figure 1, sensors are arranged on the front and rear sides of the bridge pier in the transverse direction, with eight sensors on each side, numbered i=1,2,⋯8. In this paper, cointegration is performed on two sensors at the same horizontal height.

Traditional methods generally use video cameras to monitor the impact location on the bridge. However, these methods have certain blind spots and issues with accuracy. This paper introduces the concept of the cointegration coefficient energy difference. Its principle is that under normal conditions, the energy variation in the cointegration coefficient between adjacent height sensors remains small, meaning that D approaches zero. However, after an impact occurs, the energy of the cointegration coefficient near the impact location undergoes a sudden change, causing D to increase significantly at the impact position. This helps determine the height of the impact area on the bridge pier during a collision. Based on the cointegration equation coefficients in Equation (3), the proposed damage index D is as follows:

(22) $$D_{i}={a_{i}}^{2}-a_{i-1}^{2}$$

where D is the proposed damage index, a denotes the cointegration coefficients $a_{1}$ and $a_{1}$, and i is the sensor position number.

### 3.3. Technological Implementation Process

**Figure 2:**
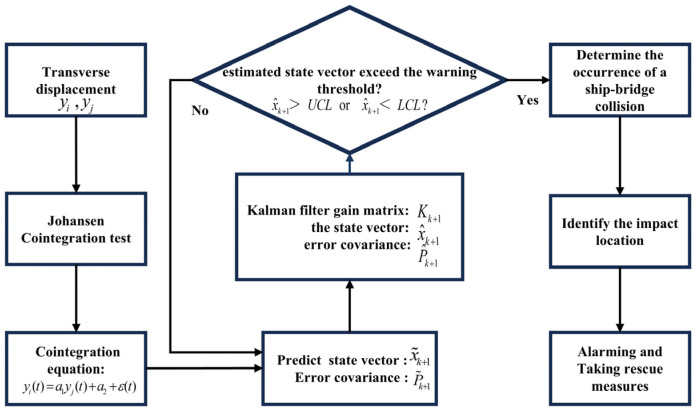
Flow chart of KFCA operation.

The implementation process of the proposed KFCA is shown in Figure 2 and consists of the following six steps:

Step 1 ADF and Johansen cointegration tests are conducted on the lateral displacement data from bridge monitoring to create the relevant cointegration equations;
Step 2 The obtained cointegration equations are used as the observation equations for the Kalman filter, and form the state vector x using the obtained cointegration coefficients $a_{1},\;a_{2}$;
Step 3 Based on Equations (16) and (17), the estimated state vector ${\hat{x}}_{k}$ and error covariance ${\hat{P}}_{k}$ from the previous time point are used to predict the state vector ${\tilde{x}}_{k+1}$ and error covariance ${\tilde{P}}_{k+1}$ at the current time point;
Step 4 Equations (18)–(20) are used to calculate the Kalman gain matrix $K_{k+1}$ from the observations obtained in Step 3, to achieve a more accurate estimate of the state vector ${\hat{x}}_{k+1}$ and error covariance ${\hat{P}}_{k+1}$;
Step 5 The latest estimates of the cointegration coefficients ${\hat{x}}_{k+1}={\left[a_{1}\;a_{2}\right]}^{T}$ are compared with the preset alarming thresholds UCL,LCL to determine whether a ship–bridge collision has occurred;
Step 6 KFCA identifies the ship–bridge collision accident, followed by the use of the damage index to locate the impacted position on the bridge.

## 4. Numerical Simulation of Ship–Bridge Collision

### 4.1. Model Descriptions

#### 4.1.1. Ship Model

**Figure 3:**
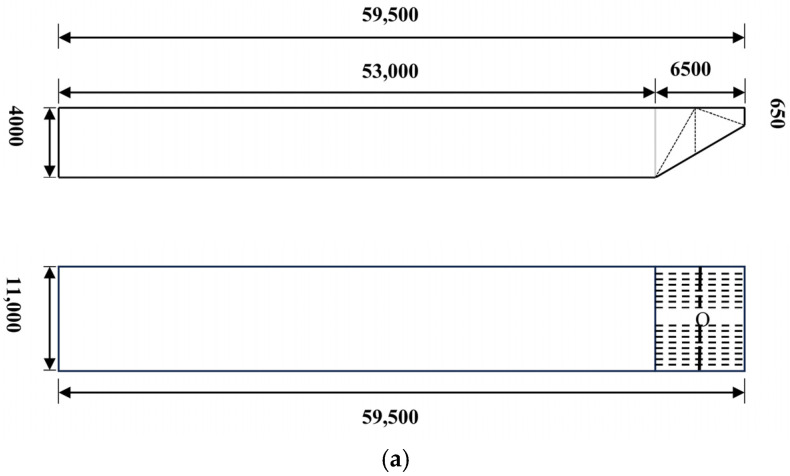

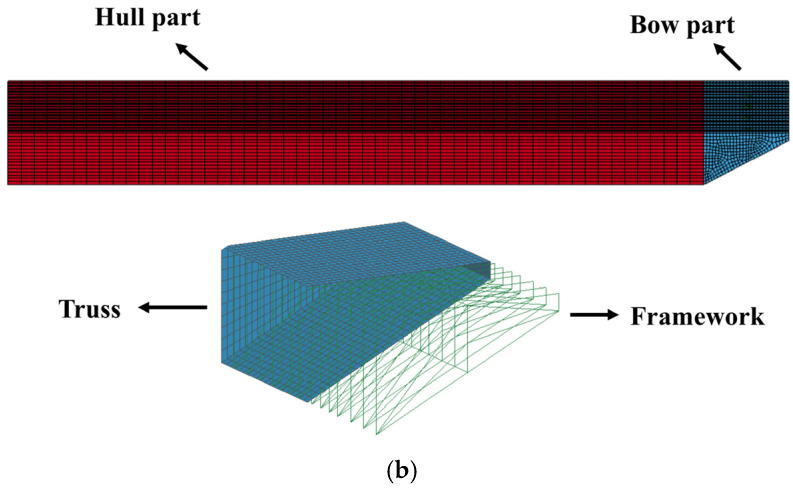
Ship modeling. (**a**) Geometric dimensions of the ship (unit: mm). (**b**) Finite element modeling of the ship.

The large hopper barge model described in the American AASHTO specification [35] is adopted in this study. The model is composed of a bow part and a hull part. During a ship–bridge collision, the bow part of the ship is assumed to experience buckling and crushing. The bow section includes structures such as outer plates, a deck, and framework. The ship model is discretized by the finite element method. The bow’s outer plate is represented by shell elements, with the thickness set to 25 mm. The truss inside the bow is modeled using beam elements, with a rectangular cross-section of 35 × 15 mm. The hull part of the ship is modeled using solid elements. The geometric dimensions and discretization model of the ship model are shown in Figure 3. The model of the ship comprises 30,158 nodes and 26,154 elements.

#### 4.1.2. Bridge Model

**Figure 4:**
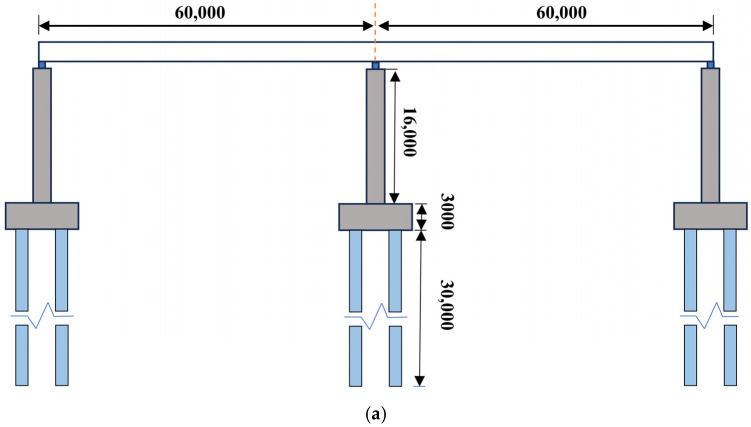

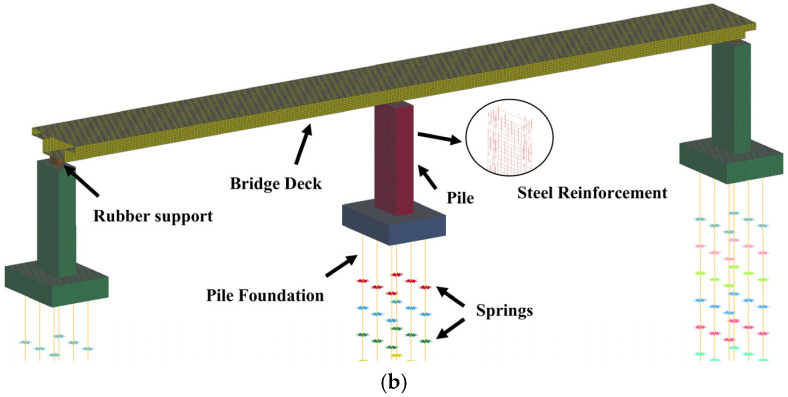
Bridge modeling. (**a**) Geometric dimensions of the bridge (unit: mm). (**b**) Finite element model of the bridge.

The bridge model is a two-span continuous beam bridge model with a total length of 120 m, consisting of the superstructure, rubber bearings, reinforced concrete piers, bridge platform, and pile foundations. The diameters of longitudinal reinforcement and stirrups are 30 mm. The embedment depth of the longitudinal reinforcement is 250 mm. The spacing of longitudinal reinforcement and stirrups is 250 mm and 400 mm, respectively. Reinforcement and concrete are coupled through nodes without consideration of the slip effect [36]. The height of the abutment is 3 m, with a length of 10 m along the bridge and 13 m across. The diameter and length of piles are 2 m and 30 m, respectively. The length of all the piles buried in the ground is 26 m. The superstructure of the bridge is modeled with shell elements, the reinforcement is modeled with beam elements, and other parts are modeled with solid elements. The detailed dimensions and modeling process of the bridge model are shown in Figure 4. The model of the bridge comprises 134,669 nodes and 113,324 elements.

The mesh quality directly affects the accuracy of finite element simulations by controlling hourglass energy in the collision system. This study evaluates three mesh sizes for bridge concrete: 100 mm, 250 mm, and 400 mm. The results show that the 400 mm mesh underestimates impact forces and dynamic responses, while the 100 mm and 250 mm meshes yield similar results. However, the 100 mm mesh significantly increases computational time. Therefore, a 250 mm mesh is selected to balance efficiency and accuracy in the barge–bridge collision simulation.

### 4.2. Constitutive Relations

In the ship’s finite element model, the steel for the bow outer plate and inner trusses is represented by the plastic kinematic constitutive model (*MAT_PLASTIC_KINEMATIC). This model accounts for the material’s strain rate and is suitable for metals subjected to explosive or impact forces. The parameters C and P are set to 40 and 5, respectively [37]. The hull section is represented by rigid material (*MAT_RIGID) with a density of 8.64 × 10^−6^ kg/mm^3^. In the ship–bridge collision simulation, 0.038 of the ship mass is considered as additional water mass [38,39], which is adjusted by modifying the hull density. The concrete used for the piers, bridge platform, and pile foundations of the impacted bridge has a strength of C40. The HJC model (*MAT_HOLMQUIST_JOHNSON_COOK) is employed to describe the concrete’s mechanical behavior under high strains and strain rates, making it suitable for impact analysis. The upper structure and additional bridge piers use a linear elastic model (*MAT_ELASTIC) with a density of 2.5 × 10^−6^ kg/mm^3^, an elastic modulus of 200 GPa, and a Poisson’s ratio of 0.3. The material for the rubber bearings is modeled as linear elastic with a density of 1.18 × 10^−6^ kg/mm^3^ and a Poisson’s ratio of 0.49. See Table 1.

### 4.3. Contact Setting

In the ship–bridge collision finite element model, two types of contact are introduced to avoid penetration between the ship and bridge during the collision and to mitigate excessive sliding energy [40]. Face-to-face contact [41] is used between the bow of the barge and the bridge pier, with the bow serving as the contact surface and the pier as the target surface. The dynamic friction coefficient is 0.3, and the static friction coefficient is 0.28. Single-sided contact is implemented between the inner truss and the outer plate of the ship’s bow, with both dynamic and static friction coefficients equal to 0.3.

### 4.4. Pile–Soil Effect

In addition, the interaction between the soil and the pile foundation must not be overlooked in a ship–bridge collision. Currently, three commonly used approaches are the direct pile–soil contact method, the equivalent soil spring method [42], and the equivalent pile diameter method [43]. In this study, the equivalent soil spring method is used to model the pile–soil interaction, and the “m-method soil spring model” is used to calculate the pile–soil interaction forces.

The spring model assumes that the soil is an elastically deformable medium, with the foundation coefficient increasing proportionally with soil depth. Therefore, the stiffness value of each horizontal soil spring layer is calculated as it increases with depth:

(23) $$k=msb_{0}h_{0}$$

where m is the horizontal resistance coefficient, set to 5000 in this paper, s is the depth of the soil from the ground surface, $b_{0}$ is the equivalent width of the pile foundation, and $h_{0}$ is the thickness of the soil.

### 4.5. Load Setting

During its service life, a bridge will endure various types of loads, such as static water pressure, vehicle loads, and wind loads. In this study, random transverse wind loads are applied to the entire bridge. According to the reference wind speed table for a specific location in China, the reference wind speed is set at 20 m/s. The static gust wind speed can be obtained from the following formula:

(24) $$V_{g}=G_{V}V_{Z}$$

where $V_{g}$ is the static gust wind speed, $G_{V}$ is the static gust wind coefficient, taken as 1.26 in this case, and $V_{Z}$ is the wind speed.

**Figure 5:**
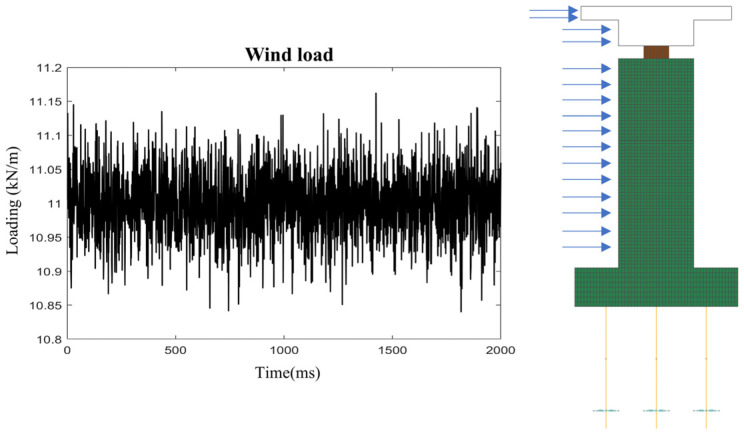
Wind loads applied to bridges.

The static wind load on the bridge under transverse wind action can be determined using the formula below, with a segment of random excitation taken at an average wind load of 11.4 kN/m, as shown in Figure 5.

(25) $$F_{H}=\frac{1}{2}\rho {V_{g}}^{2}C_{H}A_{n}$$

where $C_{H}$ denotes the drag coefficient, taken as 1.1 here, $A_{n}$ is the projected area in the wind direction, and ρ is the air density, assumed to be 1.25.

## 5. Algorithm Validation

### 5.1. Ship–Bridge Collision Alarming

#### 5.1.1. Method Validation

**Figure 6:**
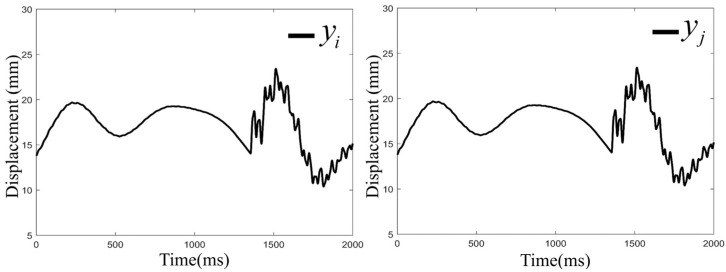
Displacement of the pier collected by the sensors.

**Figure 7:**
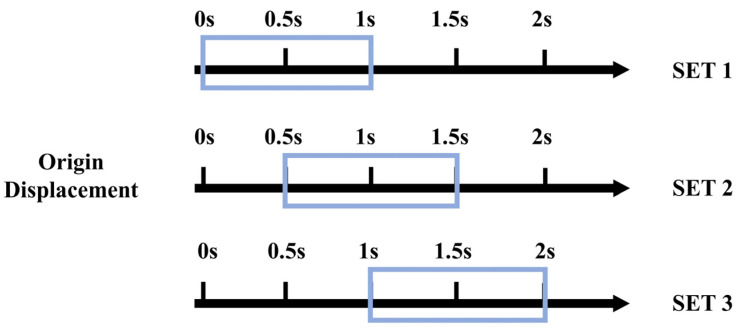
Sliding window segmentation.

To validate the effectiveness of KFCA, this paper configures the following simulation conditions: A 1000 DWT ship collides head-on with a bridge pier at a speed of 2 m/s, with a simulation time of 2000 ms and a sampling frequency of 500 Hz. The ship impacts the bridge pier at the time point of 1400 ms, causing damage to the bridge. Figure 6 shows the time series data of the transverse displacement of the bridge collected by two sensors. To monitor the bridge’s status in real time, this paper employs a sliding window method with a window size of 1 s for calculations. Each sliding window moves with a step size of 0.5 s, updating every second to guarantee immediate data processing. Specifically, the sliding windows are categorized as SET 1 (0–1 s), SET 2 (0.5–1.5 s), and SET 3 (1–2 s). At the end of each window, the sensor data are immediately fed into the warning algorithm for processing, significantly enhancing the system’s real-time response capability. Through this method, the system can maintain a high level of real-time performance, allowing for rapid detection of and response to dynamic events. The detailed configuration of the sliding window is shown in Figure 7.

Additionally, the displacement data collected by the sensors need to undergo an ADF test to determine whether they have the same order of integration. An example using data from a 2 s interval is provided below. Table 2 lists the ADF test results for the displacement data of the two sensors over 2 s. At the 1% significance level, the trace statistics for both time series are evidently below the critical values, suggesting that both series are first-order integrated, I(1), and thus have the potential for cointegration. Additionally, a Johansen cointegration test was conducted on the time series to assess the presence of a cointegration relationship, with results provided in Table 3.

Based on the results in Table 3, the Johansen test shows that the probability of no cointegration relationship between the two time series is 0, while the probability of one cointegration relationship is 93.91%. Therefore, it can be concluded that there is indeed a cointegration relationship between the displacement time series at points a and b, allowing for the use of the methods proposed in this paper to estimate the dynamic changes in this cointegration relationship. KFCA introduces an adaptive decay factor and is compared with the method without the decay factor, as shown in the results below.

**Figure 8:**
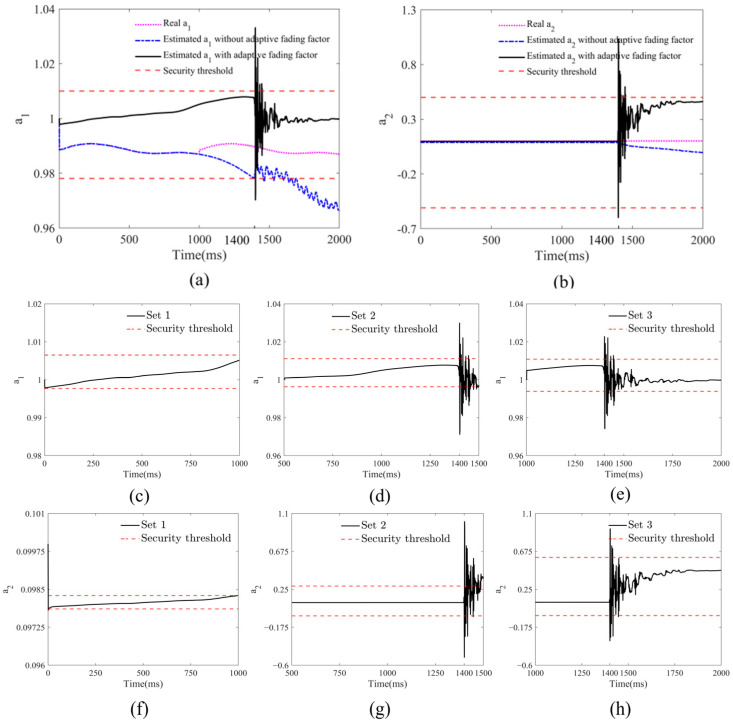
Cointegration coefficient estimation curves: (**a**,**b**) are the estimated curves for 2 s; (**c**–**e**) and (**f**–**h**) are the estimated curves for a1 and a2 under sliding window.

The pink dashed line in Figure 8 represents the cointegration coefficient estimation curve for a healthy bridge in its normal operating condition, the blue solid line represents the estimation curve using KFCA without the adaptive decay factor, and the black solid line represents the estimation curve with the adaptive decay factor introduced. Figure 8 shows that under the normal operating conditions of a healthy bridge, the monitoring data from the two sensors remain stable, and the cointegration coefficient curve becomes more level. However, during a ship–bridge collision, the ship’s impact, as an external shock load, disrupts the original stable relationship. The cointegration coefficient estimated shows a significant jump at the moment and during the process of ship impact, with its magnitude significantly exceeding the safety threshold, demonstrating its effectiveness in identifying the moment of the ship–bridge collision.

To validate the real-time performance of the KFCA proposed in this paper, we sequentially estimated the cointegration coefficients for three sliding windows: SET 1, SET 2, and SET 3. As shown in Figure 8c,f, no ship collision occurs within 0–1000 ms. Consequently, the estimated cointegration coefficients in window SET 1 develop smoothly and remain within the safe range. The ship collision moment set in this paper is at 1400 ms. As shown in Figure 8d,g, the estimated cointegration coefficients in window SET 2 develop relatively smoothly between 500 ms and 1400 ms, with no ship collision occurring. The cointegration coefficient shows a large jump at 1400 ms and exceeds the safety threshold, indicating that the ship collision occurs at 1400 ms. At this time, there is only a 100 ms delay from the actual time point of 1500 ms, demonstrating the high real-time performance of KFCA. As shown in Figure 8e,h, the estimated cointegration coefficient in window SET 3 breaches the safety limit at the 1400 ms mark, identifying the ship collision time, which corroborates the estimation results from window SET 2. Following the ongoing impact of the ship collision as an external shock load, the estimated cointegration coefficients fluctuate continuously between 1400 ms and 1700 ms, eventually stabilizing. The calculation results from the aforementioned sliding windows successfully demonstrate the high real-time performance and stability of KFCA.

#### 5.1.2. Effect of the Initial Kinetic Energy of the Ship

**Figure 9:**
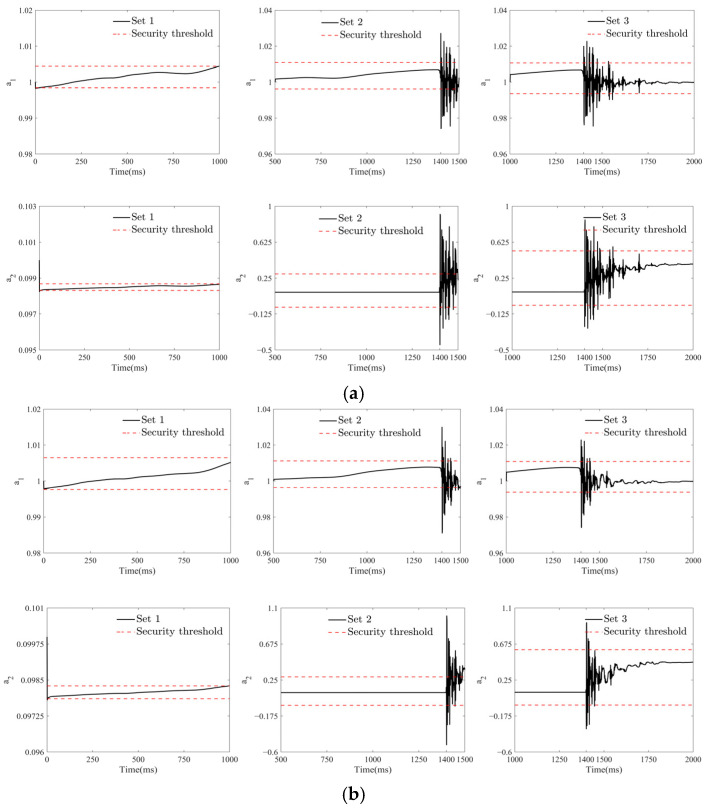

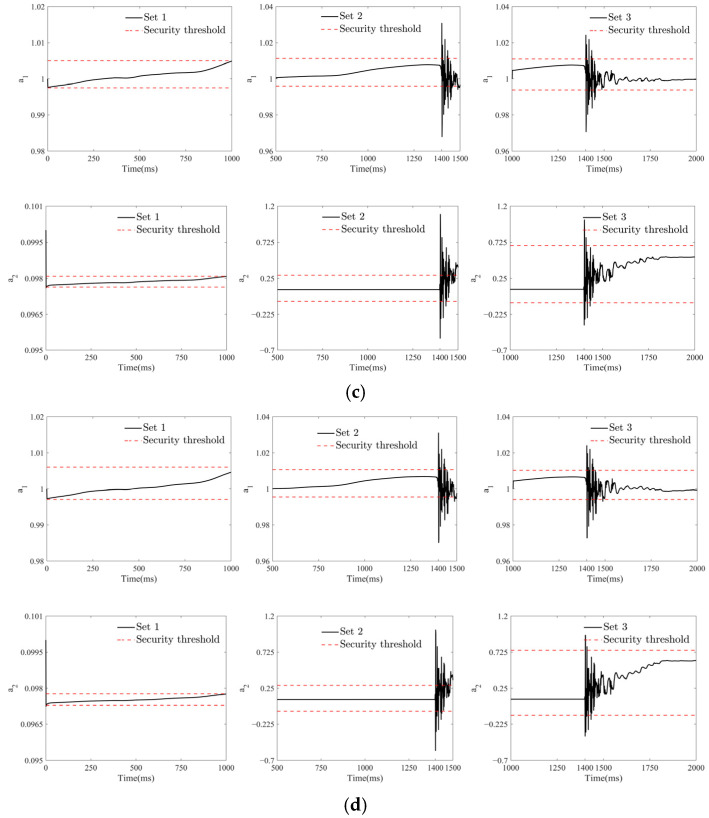
Cointegration coefficient estimation curves for different ship loads. (**a**) Cointegration coefficient estimation curve for a ship load of 567 DWT. (**b**) Cointegration coefficient estimation curve for a ship load of 1000 DWT. (**c**) Cointegration coefficient estimation curve for a ship load of 1500 DWT. (**d**) Cointegration coefficient estimation curve for a ship load of 2000 DWT.

During ship–bridge collisions, the impact velocity and load of the ship alter its initial kinetic energy, which significantly affects the collision outcome and the alarm system. Higher kinetic energy results in greater impact force, more significant structural responses, and longer impact durations, imposing stricter demands on the sensitivity and real-time performance of the alarm system. Therefore, to achieve precise alarms, it is essential to systematically examine the effect of the ship’s initial kinetic energy on the alarm algorithm to enhance adaptability to various collision scenarios. This section focuses on the performance of the alarm algorithm for varying ship loads, with ships of 567, 1000, 1500, and 2000 DWT set to impact the bridge pier at a velocity of 2 m/s. See Figure 9.

**Figure 10:**
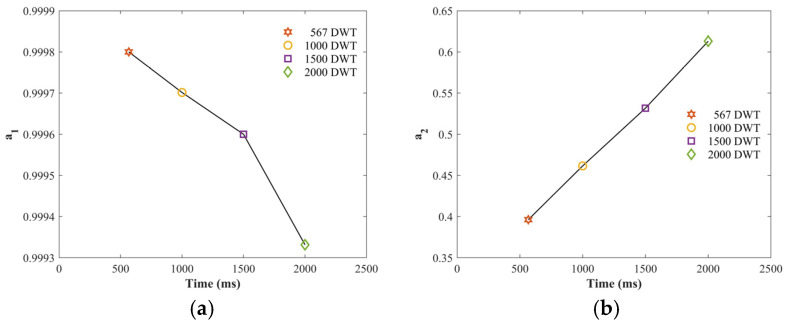
Convergence values of covariance coefficients after impacts under different loads. (**a**) Line of variation of covariance coefficient a_1_ for different ship loads. (**b**) Line of variation of covariance coefficient a_2_ for different ship loads.

The trends in the cointegration coefficient convergence curves under different ship loads are almost identical, with a significant sudden change occurring during the impact. A real-time alarm for ship–bridge collisions is successfully realized, proving that the algorithm has applicability for ship–bridge collision alarms within a certain range. This paper takes into consideration the analysis of convergence values. As shown in Figure 10, for the cointegration coefficient, the convergence value decreases as the ship’s load increases, showing an approximately linear relationship; for the cointegration coefficient, the convergence value increases as the ship’s load increases, demonstrating a linear relationship.

#### 5.1.3. Effect of Noise on Real-Time Alarming

The influence of noise was not considered in the previous section, but in practical engineering, sensor data often carry a certain amount of noise. Therefore, this section will further consider the impact of measurement noise on KFCA. In MATLAB (R2022a), the signal-to-noise ratio (SNR) can be used to add measurement noise to time series data. The SNR is typically defined as the ratio of signal power to noise power and is usually expressed in decibels (dB). The formula is shown below:

(26) $$SNR=10\times \log_{10}\left(\frac{P_{signal}}{P_{noise}}\right)$$

where $P_{signal}$ denotes the power of the original signal, and $P_{noise}$ denotes the noise power.

**Figure 11:**
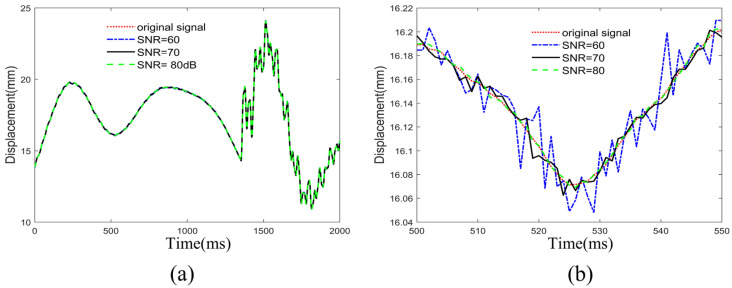
The displacement curve with noise. (**a**) Noisy graph of the complete signal. (**b**) Noisy graph of the local signal.

Figure 11a shows the displacement signals at point b after adding noise with different SNRs, with SNR values of 60, 70, and 80 dB, respectively. To better illustrate the effect of the noise, Figure 11b provides a magnified view of the signal within the time interval from 500 to 550. In Figure 11, the red dashed line represents the original signal, the blue solid line represents the signal with an SNR of 60 dB, the black solid line represents the signal with an SNR of 70 dB, and the green solid line represents the signal with an SNR of 80 dB.

**Figure 12:**
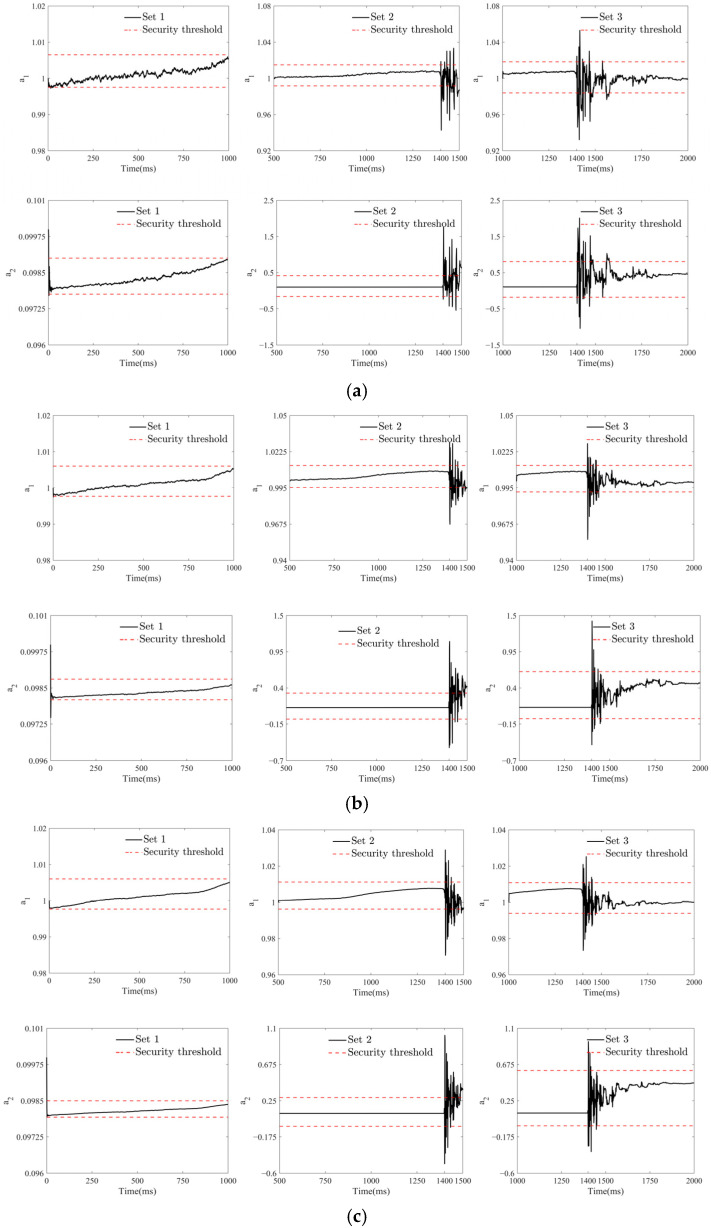
Cointegration coefficient estimation curve with noise. (**a**) Cointegration coefficient estimation curve under noise with SNR of 60 dB. (**b**) Cointegration coefficient estimation curve under noise with SNR of 70 dB. (**c**) Cointegration coefficient estimation curve under noise with SNR of 80 dB.

Figure 12 shows that the roughness of the cointegration coefficient estimation curves in each time window increases as the SNR decreases, which is particularly evident in the no-ship-collision window SET 1. After the ship collision occurs, the cointegration coefficient estimation curve under an SNR of 60 dB exhibits the greatest fluctuation and the poorest convergence performance. Nevertheless, as shown in window SET 2 of Figure 12a–c, the first time at which the cointegration coefficient estimation curve of the warning algorithm crosses the safety threshold is 1400 ms. After this, there are still some instances where the safety threshold is exceeded, but these all occur within the duration of the ship–bridge collision. Therefore, it can be concluded that KFCA is capable of accurately identifying the ship–bridge collision time under different noise conditions, demonstrating excellent noise resistance.

#### 5.1.4. Comparison with the Wavelet Transform Technology

The wavelet transform is an advanced signal processing method that breaks down signals into various frequency components to examine their time and frequency attributes. This process generates approximation and detail signals, where the approximation signal represents the overall trend of the signal, and the detail signal captures the local features of the signal. This paper introduces wavelet transform technology and compares it with KFCA to evaluate their respective performances and advantages. This comparison is intended to validate the efficacy of the wavelet transform in extracting signal features and identifying ship–bridge collision moments, and to highlight the advantages of KFCA in accurately detecting these moments.

**Figure 13:**
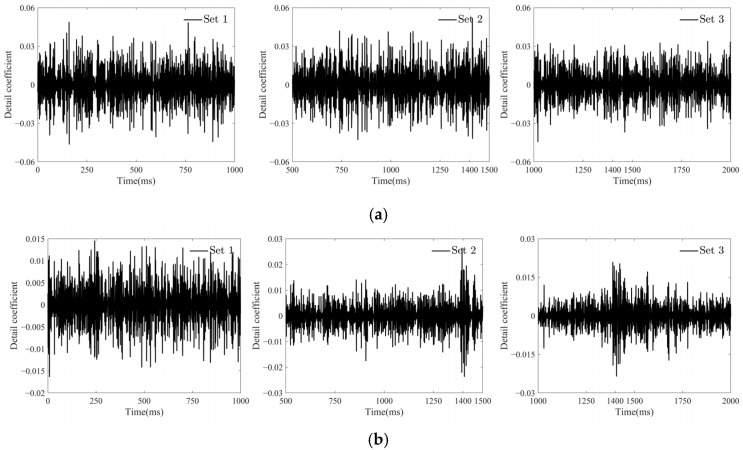

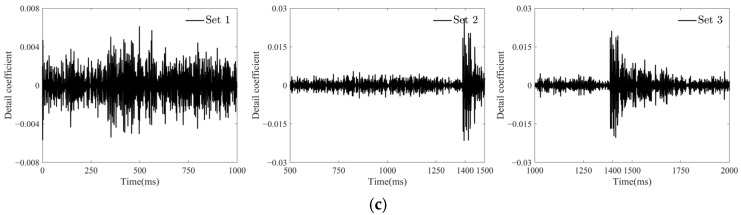
Detail signals from a wavelet transform within a sliding window. (**a**) Wavelet transform detail signals for SNR of 60 dB. (**b**) Wavelet transform detail signals for SNR of 70 dB. (**c**) Wavelet transform detail signals for SNR of 80 dB.

As shown in Figure 13c, at an SNR of 80 dB, the detail signal of the wavelet transform exhibits a significant jump at the time of the ship collision (around 1400 ms) and then gradually returns to normal. This indicates that the wavelet transform can effectively extract features of the ship collision signal, thereby accurately identifying the time of the collision. Figure 13b shows that at an SNR of 70 dB, the wavelet transform detail signal exhibits a subtle jump at 1400 ms, but the detail signal is relatively coarse at this time. When the SNR drops to 60 dB, as shown in Figure 13c, the detail signal of the wavelet transform becomes very chaotic, making it impossible to identify the ship–bridge collision time. This indicates that in a high-noise environment, relying solely on the wavelet transform makes it difficult to accurately extract features of the ship collision signal. As shown in the results in Figure 12 and Figure 13, compared to the wavelet transform method, the KFCA proposed in this paper demonstrates higher robustness under the same noise levels. In conclusion, KFCA demonstrates significant superiority and stability in ship collision warning systems, maintaining good recognition performance in complex environments.

### 5.2. Impact Location Identification

#### 5.2.1. Effect of Impact Location

**Figure 14:**
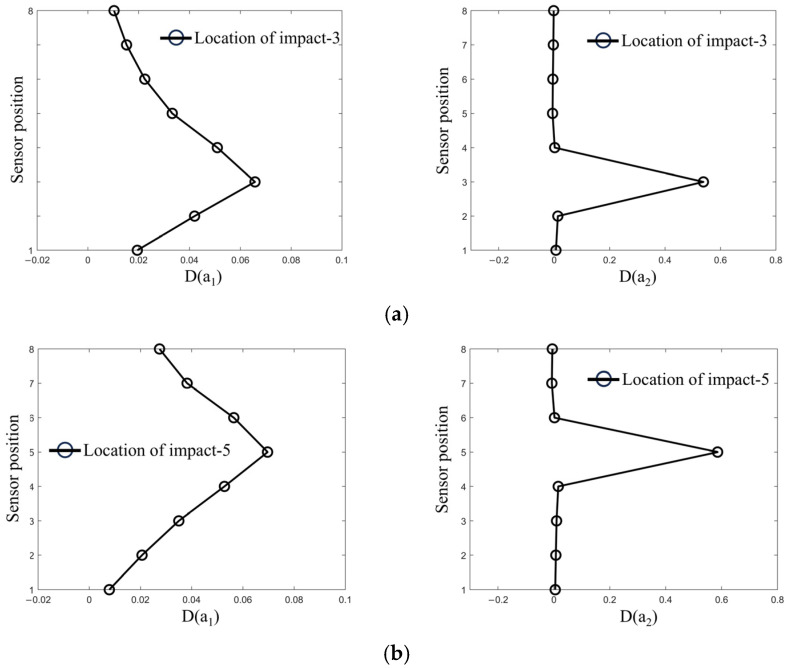

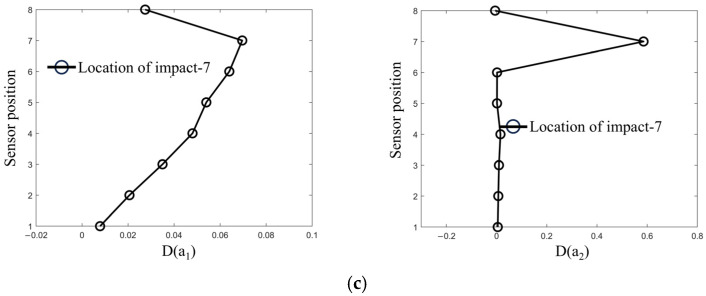
Cointegration coefficient energy differences at various impact locations. (**a**) Cointegration coefficient energy difference at impact point 3. (**b**) Cointegration coefficient energy difference at impact point 5. (**c**) Cointegration coefficient energy difference at impact point 7.

The simulation conditions are set with a 1000 DWT ship colliding head-on with the bridge pier at a speed of 2 m/s, with a simulation time of 2 s, a sampling frequency of 500 Hz, and impact locations set near a horizontal height of i=3, 5, 7. As shown in Figure 14a, after the ship–bridge collision, the cointegration relationship of the sensor data collected at the impact height i=3 changes dramatically. Accordingly, a significant change in the cointegration coefficient energy difference D at this height indicates that the bridge pier at height i=3 is impacted by the ship. In Figure 14b,c, when the impact location is at i=5, 7, the proposed identification index effectively determines the impact location. These results validate the reasonableness of the proposed index.

#### 5.2.2. Effect of Noise on Impact Location Estimation

This section will further consider the effect of measurement noise on the accuracy of the proposed index D for determining the impact location. In this section, measurement noise will be introduced to the time series data using SNRs of 20 dB, 40 dB, and 60 dB, with the impact location set at a horizontal height of i=5 .

**Figure 15:**
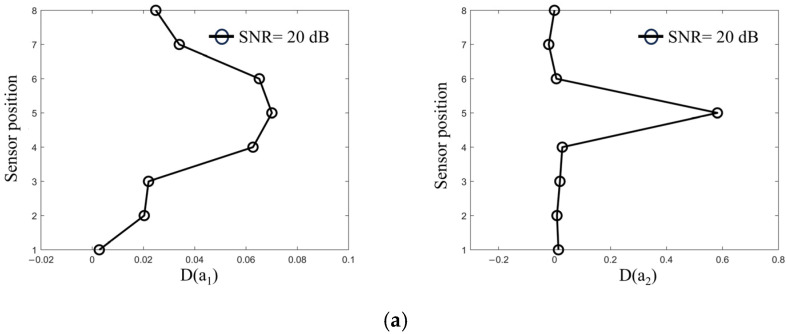

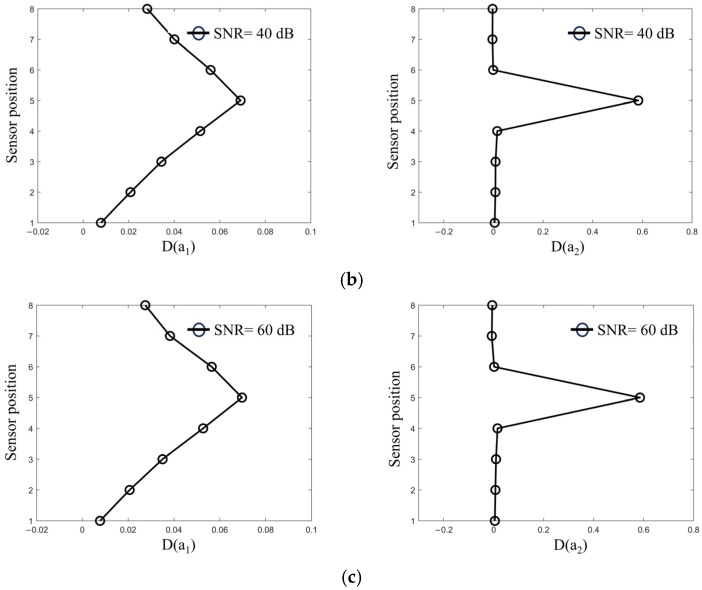
Cointegration coefficient energy differences at various impact locations. (**a**) Cointegration coefficient energy difference at SNR = 20. (**b**) Cointegration coefficient energy difference at SNR = 40. (**c**) Cointegration coefficient energy difference at SNR = 60.

As shown in Figure 15, the $D(a_{1})$ cointegration coefficient energy difference decreases progressively from the impact point i=5 toward locations farther from the impact, with a significant jump occurring near the impact location. However, the change in $D(a_{1})$ at an SNR of 20 dB is notably less smooth, suggesting that it is relatively more sensitive to noise. On the other hand, as shown in Figure 15, the index $D(a_{2})$ corresponding to the cointegration coefficient $a_{2}$ is relatively insensitive to noise variations, with only minor fluctuations observed at an SNR of 20 dB. This indicates that the index remains stable even at higher noise levels. In conclusion, the proposed ship impact bridge position identification index can accurately identify the location of ship impacts on bridge piers across different noise levels, exhibiting strong noise resilience and significant application potential.

## 6. Discussion

In this paper, the effects of the water body and pile–soil interaction were simplified, and a full bridge finite element model of the ship–bridge system was established. It was assumed that the soil provides only lateral constraints on the pile foundation and that the sensors function properly to collect reliable data. Under these conditions, a time series analysis method based on the Kalman filter–cointegration was adopted to identify the exact moment of ship–bridge collision. Furthermore, a novel damage index D, the cointegration coefficient energy difference, was proposed to determine the collision location. The main conclusions are as follows:

(1) The KFCA in this paper effectively captures the response of bridges under ship impact disturbances during normal operations. By importing the response data into alarming technology using a sliding window approach, it successfully identifies ship–bridge collision. The estimation results from different windows demonstrate that KFCA has strong real-time performance;
(2) This paper investigates the impact of ships with varying load scales on bridge collisions and the identification method proposed. The study found that KFCA can identify and provide real-time alarms for collisions caused by multi-scale-load ships, demonstrating the broad applicability potential of KFCA.
(3) This paper explores and compares the performance of KFCA and the wavelet transform method under different noise levels. The results show that KFCA demonstrates significant superiority and stability in ship–bridge collision alarming technology, maintaining good performance in complex environments.
(4) Based on the identification of the occurrence of ship–bridge collision, the damage index proposed in this paper amplifies the changes in cointegration coefficients at multiple locations during the collision event. It successfully identifies the impact area location under different impact locations and noise levels, offering useful insights for evaluating ship–bridge collisions.

Although KFCA can identify ship–bridge collisions in real time and demonstrates strong stability, it still has the following limitations: its application in extreme environments requires further validation and improvement; and its ability to detect multiple consecutive collisions needs further verification. Future developments can focus on integrating multi-source sensor data, optimizing adaptive parameter adjustment mechanisms, and extending its application to more complex vessel–bridge collision scenarios to enhance its applicability and accuracy.

## Figures and Tables

**Table 1:** Material parameters.

Structure	Models in LS-DYNA	Material Parameters
Hull	*MAT_RIGID	Density: variable Young’s modulus: 200 Gpa Poisson’s ratio: 0.3
Upper structure	*MAT_ELASTIC	Density: 2.5 × 10^−6^ kg/mm^3^ Young’s modulus: 200 Gpa Poisson’s ratio: 0.3
Rubber support	*MAT_ELASTIC	Density: 1.18 × 10^−6^ kg/mm^3^ Young’s modulus: 200 Gpa Poisson’s ratio: 0.49
Outer plate Trusses	*MAT_PLASTIC_KINEMATIC	Density: 7.89 × 10^−6^ kg/mm^3^ Young’s modulus: 200 Gpa Poisson’s ratio: 0.3 C: 40 P: 5
Pile foundations Bridge platform	*MAT_HOLMQUIST_JOHNSON_COOK	Density: 2.5 × 10^−6^ kg/mm^3^ Shear modulus: 14.51 Gpa

**Table 2:** ADF test results.

First Difference	Critical Value			t Statistic
	1%	5%	10%	ADF Test Results
$\Delta y_{i}$	−3.432	−2.862	−2.567	−17.417
$\Delta y_{j}$	−3.432	−2.862	−2.567	−18.058

**Table 3:** Johansen cointegration test results.

Existence of Cointegration Relationship	Eigenvalue	Trace Statistic	0.05 Critical Value	Prob.
None	0.03378	124.9322	25.87211	0.0000
At most 1	0.00134	4.704910	12.51798	0.9391

## Data Availability

Data are unavailable due to privacy restrictions.
